# Identification of HMOX-1-Targeting Natural Compounds in *Camellia nitidissima* Chi for NSCLC Therapy: Integrating Bioassay and In Silico Screening Approaches

**DOI:** 10.3390/ph18060824

**Published:** 2025-05-30

**Authors:** Lingqiu Zhang, Fan Zhang, Haimei Liang, Xiangling Qin, Chunmei Liang, Manlu Zhong, Yuemi Mo, Jinling Xie, Xiaotao Hou, Jiagang Deng, Erwei Hao, Zhengcai Du

**Affiliations:** 1 Guangxi Key Laboratory of Efficacy Study on Chinese Materia Medica, University Engineering Research Center of Reutilization of Traditional Chinese Medicine Resources, Guangxi University of Chinese Medicine, Nanning 530200, China; zhanglingqiu2022@stu.gxtcmu.edu.cn (L.Z.); zhangf@gxtcmu.edu.cn (F.Z.); lianghaimei2022@stu.gxtcmu.edu.cn (H.L.); qinxiangling2023@stu.gxtcmu.edu.cn (X.Q.); lcm277917@126.com (C.L.); w2685462394@126.com (M.Z.); moyuemi2023@stu.gxtcmu.edu.cn (Y.M.); 13257716536@163.com (J.X.); xthou@126.com (X.H.); dengjg53@126.com (J.D.); 2 Guangxi Key Laboratory of TCM Formulas Theory and Transformation for Damp Diseases, Institute of Traditional Chinese and Zhuang-Yao Ethnic Medicine, Guangxi University of Chinese Medicine, Nanning 530200, China; 3 International Zhuang Medical Hospital, Guangxi University of Chinese Medicine, Nanning 530000, China

**Keywords:** *Camellia nitidissima* leaves, ferroptosis, NSCLC, bioactive screening, preparative separation

## Abstract

**Background/Objectives:** *Camellia nitidissima* Chi (*C. nitidissima*), a traditional Chinese “food and medicine homology” plant, has demonstrated potential anti-tumor properties. However, its mechanisms of anti-lung cancer activity via ferroptosis remain unclear. This study aimed to construct an integrated research system of “natural product extraction-purification, bioactivity evaluation, and computational drug screening” to explore the bioactive compounds in *C. nitidissima* leaves targeting HMOX-1-mediated ferroptosis and their anti-lung cancer mechanisms. **Methods:** Active fractions were prepared using ethanol extraction combined with polyamide column chromatography. The anti-lung cancer activity was evaluated using the NCI-H1975 cell model. Ferroptosis was verified via transmission electron microscopy (TEM), biochemical indicators, a PCR Array, and immunofluorescence. The bioactive compounds were identified using UPLC-Q Exactive MS, and their binding affinity to HMOX-1 was evaluated via molecular docking and dynamics simulations, followed by cellular validation. **Results:** The 95% F1 fraction from the extracts of *C. nitidissima* leaves exhibited the strongest anti-lung cancer activity, which could be significantly reversed by Ferrostatin-1. Furthermore, it induced typical ferroptosis-related structural damage in mitochondria, including shrinkage and a reduction in size, increased membrane density, and a reduction or even the disappearance of cristae structures. At the molecular level, this fraction significantly increased the levels of oxidative stress markers (ROS↑, MDA↑, Fe^2+^↑, and GSH↓) and upregulated the expression of key ferroptosis-related genes, including HMOX-1, CHAC1, and NOX1. Using UPLC-Q Exactive MS combined with computational simulation methods, four bioactive compounds with high affinity for HMOX1 were successfully identified, including isochlorogenic acid A (−8.4 kcal/mol), isochlorogenic acid C (−8.4 kcal/mol), apigenin (−7.8 kcal/mol), and chrysin (−7.3 kcal/mol). Cellular experiments validated that these compounds exhibited dose-dependent anti-proliferative effects. **Conclusions:** The leaves of *C. nitidissima* induce anti-lung cancer effects via HMOX-1-mediated ferroptosis. Isochlorogenic acid A/C, apigenin, and chrysin were identified as key bioactive components. These findings lay the foundation for the development of natural ferroptosis-targeted drugs.

## 1. Introduction

Lung cancer is among the malignancies with the highest incidence and mortality rates worldwide, with non-small cell lung cancer (NSCLC) accounting for approximately 85% of all lung cancer cases [1]. Despite continuous advancements in various treatment modalities, including surgery, radiotherapy, chemotherapy, targeted therapy, and immunotherapy, the prognosis for lung cancer patients remains poor, with a 5-year survival rate of merely 16% [2]. This grim scenario has prompted researchers to seek novel therapeutic targets and innovative strategies to overcome current treatment bottlenecks. Recently, ferroptosis has attracted considerable attention as a novel form of programmed cell death due to its unique molecular mechanisms and therapeutic potential [3,4]. Ferroptosis is primarily characterized by significantly elevated intracellular levels of iron ions and reactive oxygen species (ROS), leading to lipid peroxidation and subsequent cell membrane damage [5,6]. Unlike traditional apoptosis and necrosis, ferroptosis is regulated through specific molecular pathways, including the inactivation of glutathione peroxidase 4 (GPX4), the modulation of iron metabolism-related proteins such as heme oxygenase 1 (HMOX-1), and the activation of oxidative stress-associated signaling cascades [7,8,9]. In lung cancer, the induction of ferroptosis has been demonstrated to effectively inhibit tumor cell proliferation [10], providing promising new avenues for therapeutic interventions.

Natural products and their bioactive constituents have garnered considerable interest in anti-cancer research owing to their multi-target regulatory mechanisms and relatively low toxicities [11]. More and more studies have reported that plant-derived active ingredients are involved in regulating key pathways of ferroptosis, including regulating iron homeostasis, the antioxidant system, and lipid metabolism, thus exhibiting anti-tumor potential [12,13]. Therefore, the discovery and screening of natural ferroptosis modulators is of great significance for the development of innovative anti-cancer drugs. *Camellia nitidissima* Chi (*C. nitidissima*), belonging to the Camellia genus of the Theaceae family, is distinguished by its uniquely golden petals, earning it the titles “Queen of Tea” and “Giant Panda of the Plant Kingdom” [14,15]. Native and endemic to China, this plant has been internationally referred to as the “Magical Oriental Tea”. Its leaves have a long history of medicinal and health care applications and are rich in a variety of bioactive components such as flavonoids and polyphenols. In 2010, *C. nitidissima* was officially listed as a medicinal-edible dual-purpose resource and classified as a novel food resource, signifying significant development potential [16,17]. Previous studies have demonstrated various pharmacological activities of *C. nitidissima* extracts, including antioxidant, anti-inflammatory, and anti-tumor effects, highlighting its considerable health-promoting and therapeutic potential [18,19,20].

Although *C. nitidissima* has been traditionally used in Chinese medicine for health maintenance and as an adjunct treatment for cancer and its inhibitory effects on colon cancer have been reported [21], its anti-lung cancer mechanisms remain largely unexplored, particularly regarding ferroptosis. To date, no systematic clinical trials or approved drugs have been reported for the use of *C. nitidissima* in NSCLC treatment. Therefore, further investigation into *C. nitidissima*’s role in ferroptosis is urgently needed to uncover its molecular mechanisms and identify key active components, thus laying the groundwork for broadening its anti-cancer spectrum and clinical translational potential.

**Figure 1:**
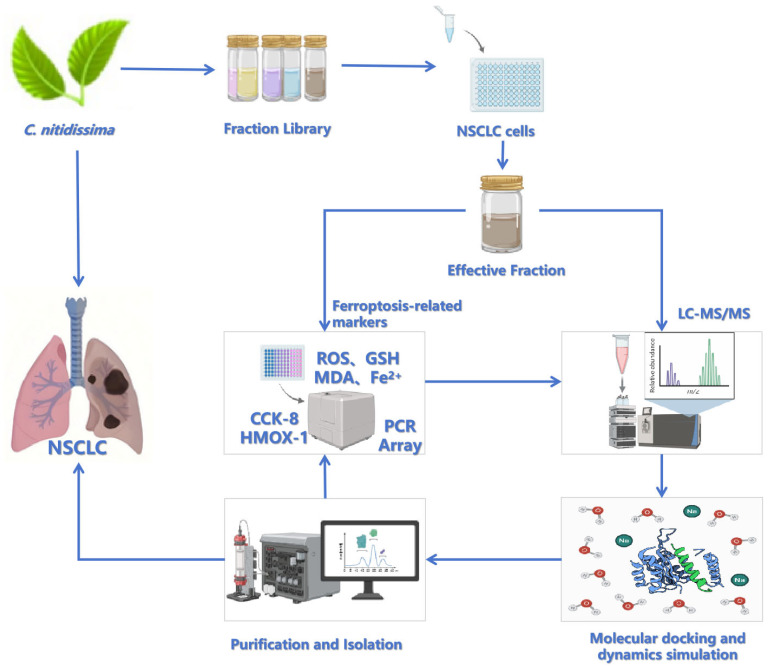
Schematic workflow of the integrated experimental design.

This study focuses on the underlying mechanisms by which *C. nitidissima* leaf extracts inhibit the growth of NSCLC cells by modulating the ferroptosis pathway. The following research strategy was employed (Figure 1): (1) Based on in vitro anti-lung cancer activity, active fractions with different polarities were isolated and screened from *C. nitidissima* leaves. (2) The ability to induce ferroptosis was systematically validated by observing the mitochondrial ultrastructure and detecting ferroptosis biomarkers. (3) The potential interactions between the active fractions and key targets, such as HMOX-1, were comprehensively elucidated through UPLC-Q Exactive-MS analysis, molecular docking studies, and molecular dynamics (MD) simulations. (4) Representative compounds were further isolated to verify their molecular mechanisms and biological activities. This integrated research strategy of “natural product screening—mechanism validation—target identification” is conducive to exploring the anti-cancer potential of Chinese characteristic plant resources and promoting the development of ferroptosis-targeted new drugs.

## 2. Results

### 2.1. Cytotoxic Assessment of C. nitidissima Leaf Ethanol Extract in NSCLC

*C. nitidissima* leaves were subjected to two successive rounds of reflux extraction using 75% ethanol, with each extraction lasting 2.0 h, totaling 4.0 h. After extraction, the solvent was removed via rotary evaporation, yielding a crude extract with an approximate yield of 13.6%. This provided a sufficient quantity of material for subsequent chromatographic purification experiments.

**Figure 2:**
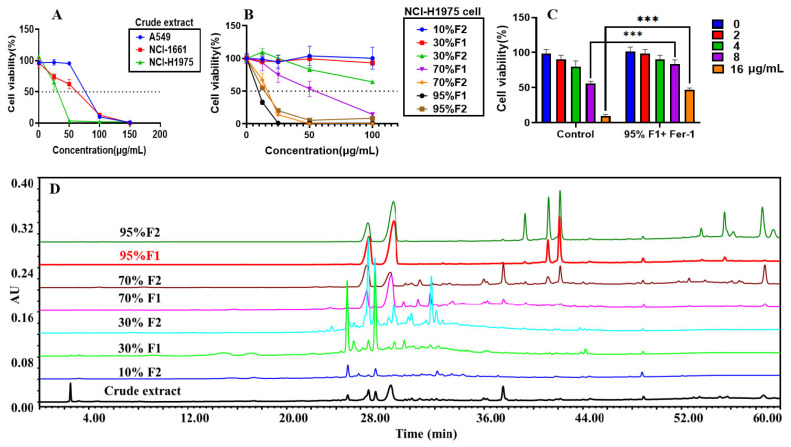
Cytotoxic effects of crude extract and polarity-based fractions of *C. nitidissima* on lung cancer H1975 cells and corresponding HPLC chromatograms. (**A**) Inhibitory effects of the *C. nitidissima* crude extract on the viability of the A549, NCI-H1661, and NCI-H1975 lung cancer cell lines. (**B**) Effects of different polarity fractions of *C. nitidissima* on the viability of NCI-H1975 cells. (**C**) Verification of ferroptosis involvement by assessing the cytotoxicity of the 95% F1 fraction in the presence of the ferroptosis inhibitor Fer-1. Results are expressed as the mean ± SEM (*n* = 4). ***: *p* < 0.0001 compared with the control group (treatment with 95% F1 alone). (**D**) HPLC fingerprint chromatograms of the crude extract and various polarity fractions of *C. nitidissima*.

To evaluate the in vitro anti-tumor activity of the ethanol extract, the sensitivity of three NSCLC cell lines—A549, NCI-H661, and NCI-H1975—was assessed following treatment with various concentrations of the extract (25 μg/mL to 150 μg/mL). Cells treated with the culture medium alone served as the controls. Among the tested cell lines, NCI-H1975 exhibited the highest sensitivity to the extract, with an IC_50_ value of 27.97 μg/mL, followed by NCI-H661 (IC_50_ = 51.84 μg/mL), while A549 was the least sensitive (IC_50_ = 74.66 μg/mL). These results indicate that the ethanol extract of *C. nitidissima* leaves effectively inhibits the growth of NSCLC cells, with the greatest cytotoxicity observed in NCI-H1975 cells. Therefore, NCI-H1975 was selected for subsequent mechanistic investigations (Figure 2A).

### 2.2. Fractionation and Purification of C. nitidissima Leaf Ethanol Extract and Bioactivity-Guided Anti-Lung Cancer Screening

This study aimed to isolate and purify the bioactive constituents from the *C. nitidissima* leaf ethanol extract using polyamide column chromatography, followed by the evaluation of their inhibitory effects on NCI-H1975 lung cancer cells. Stepwise elution was performed with ethanol solutions of increasing concentrations (10%, 30%, 70%, and 95%) as eluents, resulting in the successful separation of seven fractions with distinct polarities (Figure 2D), thereby establishing a comprehensive fraction library of *C. nitidissima* leaves (Table 1). This fractionation process provided a solid material foundation for subsequent bioactivity screening and compound identification.

Cell viability assays were conducted on the collected fractions to determine their cytotoxic effects on NCI-H1975 cells. Among the fractions, the 95% F1 fraction exhibited the most potent inhibitory activity, with an IC_50_ value of 10.95 μg/mL (Figure 2B, Table 1). Based on these findings, the 95% F1 fraction was selected for further investigation to elucidate its underlying mechanisms of action and identify its active constituents.

### 2.3. Induction of Ferroptosis in NCI-H1975 Lung Cancer Cells by the 95% F1 Fraction

#### 2.3.1. Ferrostatin-1 Reverses the Anti-Cancer Activity of 95% F1 Against NCI-H1975 Cells

To investigate whether ferroptosis is involved in the cytotoxic mechanism of the 95% F1 fraction, we examined the effect of the ferroptosis inhibitor Ferrostatin-1 (Fer-1) on 95% F1-induced cell death in NCI-H1975 cells. As shown in Figure 2C, co-treatment with Fer-1 significantly restored cell viability compared to treatment with 95% F1 alone at concentrations of 8.0 μg/mL and 16 μg/mL (*p* < 0.0001). These findings suggest that the cytotoxicity of the 95% F1 fraction is at least partially mediated by ferroptosis, and Fer-1 can effectively attenuate this effect.

#### 2.3.2. 95% F1 Induces Ferroptosis via ROS, MDA, and Fe^2+^ Accumulation and GSH Depletion

**Figure 3:**
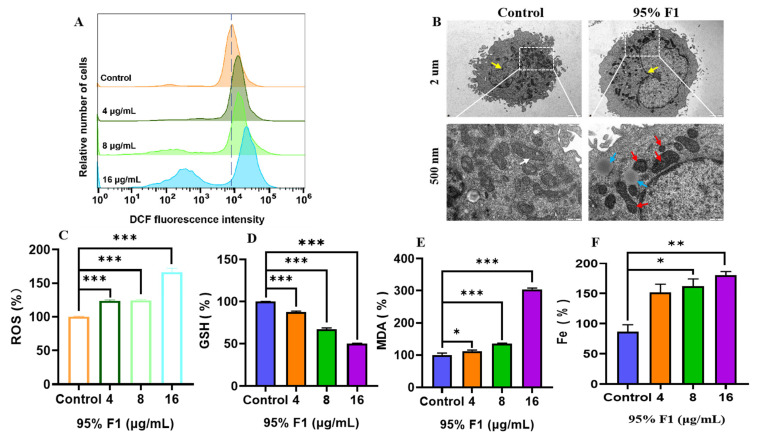
Mechanistic investigation of ferroptosis induction in H1975 lung cancer cells by the 95% F1 of *C. nitidissima*. (**A**,**C**) Flow cytometry analysis of intracellular ROS levels in H1975 cells treated with varying concentrations of the 95% F1 fraction using the DCFH-DA fluorescent probe. (**B**) Mitochondrial ultrastructure of NCI-H1975 cells compared with the control group. The mitochondrial ultrastructure in NCI-H1975 cells treated with 95% F1 (8.0 μg/mL) exhibited shrinkage and reduced size, increased membrane density, and a decrease or even disappearance of cristae structures (red arrow). Additionally, lipid droplets (blue arrow) and nuclei (yellow arrow) were observed. In contrast, normal mitochondria were abundant and evenly distributed, with clear double-membrane structures and lamellar cristae, and the cristae protrusions were arranged in a roughly parallel lamellar pattern (white arrow). (**D**–**F**) Quantitative bar graphs of cellular levels of GSH, MDA, and Fe^2+^, respectively, following treatment. Results are expressed as the mean ± SEM (*n* = 3). *: *p* < 0.05, **: *p* < 0.01, and ***: *p* < 0.0001 compared with the control group.

Flow cytometry analysis revealed that 95% F1 significantly increased intracellular ROS levels in a dose-dependent manner (Figure 3A,C). Compared to the control, DCF fluorescence intensity was markedly elevated at 4.0 μg/mL and further intensified at 8.0 and 16 μg/mL, indicating substantial ROS accumulation. Excessive ROS generation may disrupt redox homeostasis, leading to oxidative stress, mitochondrial damage, lipid peroxidation, and ultimately ferroptosis.

GSH, a key intracellular antioxidant, was found to decrease progressively with increasing concentrations of 95% F1, with a significant reduction observed at 16 μg/mL (Figure 3D). MDA, a marker of lipid peroxidation and oxidative membrane damage, exhibited a concentration-dependent increase, which was especially pronounced at 16 μg/mL (Figure 3E). Although the increase in Fe^2+^ was less marked, a notable elevation was detected at 16 μg/mL, suggesting the disruption of intracellular iron homeostasis (Figure 3F). The Fenton reaction between Fe^2+^ and ROS can generate highly reactive hydroxyl radicals (•OH), further aggravating oxidative damage—a hallmark of ferroptosis.

These findings demonstrate that 95% F1 induces ferroptosis through a concentration-dependent increase in ROS, the depletion of GSH, an elevation of MDA levels, and the disruption of iron homeostasis. The observed oxidative stress and membrane lipid damage reflect classical features of ferroptosis (ROS↑, GSH↓, MDA↑, and Fe^2+^↑), supporting the therapeutic potential of 95% F1 in targeting ferroptotic pathways in NCI-H1975 cells.

#### 2.3.3. Ultrastructural Alterations of Mitochondria Induced by 95% F1

Transmission electron microscopy revealed distinct mitochondrial changes in NCI-H1975 cells treated with 95% F1 (8.0 μg/mL) (Figure 3B). Compared with the untreated control group, where mitochondria displayed intact double membranes, densely packed cristae, and a normal morphology, cells in the treated group exhibited hallmark ferroptotic features, including mitochondrial shrinkage, increased membrane density, and a loss of or reduction in cristae. These structural changes suggest that 95% F1-induced oxidative stress may lead to mitochondrial membrane lipid peroxidation, the disruption of mitochondrial membrane potential, impaired energy metabolism, and eventual cell death. The ultrastructural data are consistent with the biochemical findings and further support the conclusion that ferroptosis is a key mechanism of action.

#### 2.3.4. The 95% F1 Fraction Induces Differential Expression of Genes Involved in Ferroptosis

**Figure 4:**
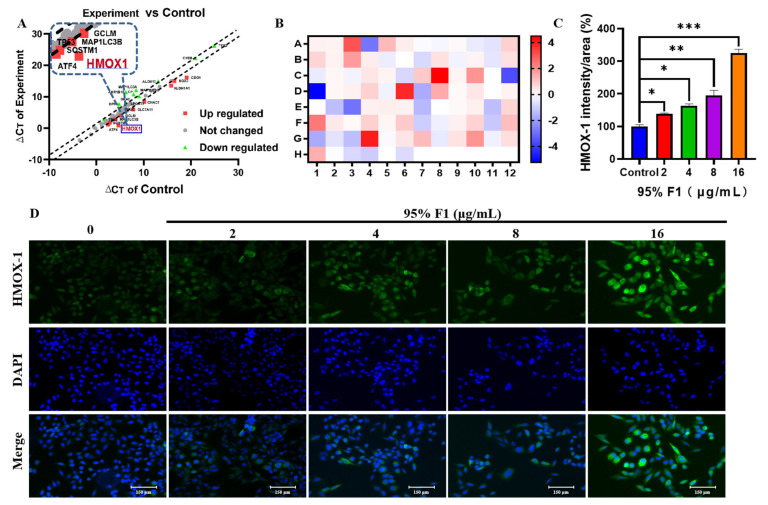
The 95% F1 fraction of *C. nitidissima* upregulates the expression of HMOX-1 to induce ferroptosis in NCI-H1975 lung cancer cells. (**A**) Differential gene expression analysis using the PCR Array, highlighting key genes associated with ferroptosis. (**B**) Heatmap of gene expression changes under different treatment conditions, demonstrating the expression profile of ferroptosis-related genes. (**C**) Effects of different treatments of 95% F1 (2, 4, 8, and 16 μg/mL) on HMOX-1 expression levels. The bar graph shows the HMOX-1 intensity/area (%) in different treatment groups compared to the control group. (**D**) Immunofluorescence images showing HMOX-1 expression levels in H1975 cells treated with increasing concentrations of the 95% F1 fraction (0, 2, 4, 8, and 16 μg/mL). Results are expressed as the mean ± SEM (*n* = 3). *: *p* < 0.05, **: *p* < 0.01, and ***: *p* < 0.0001 compared with control group.

To elucidate the molecular mechanisms underlying ferroptosis, we analyzed the expression profiles of ferroptosis-related genes in NCI-H1975 cells following treatment with the 95% F1 fraction (Figure 4A,B). Quantitative PCR analysis using the 2^−ΔΔCt^ method revealed that multiple ferroptosis-associated genes exhibited significant expression changes. Canonical ferroptosis-promoting factors, including HMOX-1, CHAC1, NOX1, NOX3, FTMT, CDO1, and MAP1LC3B, were markedly upregulated, indicating enhanced oxidative stress, intracellular Fe^2+^ accumulation, and elevated autophagic activity, suggesting a cellular state highly susceptible to ferroptosis. In contrast, key genes involved in iron homeostasis, lipid peroxidation, and antioxidant defense, such as CP, ALOX15, LPCAT3, TP53, GCLC, and MAP1LC3A, were significantly downregulated, further impairing the cells’ ability to counteract iron-dependent oxidative stress. Notably, the prominent upregulation of HMOX-1 may accelerate Fe^2+^ release and amplify lipid peroxidation chain reactions, acting as a crucial molecular trigger of ferroptosis. Collectively, these transcriptional changes provide compelling evidence that the active components within the 95% F1 fraction effectively induce ferroptotic cell death in NCI-H1975 cells by coordinately modulating iron metabolism, lipid oxidation, and redox homeostasis.

#### 2.3.5. 95% F1 Induces Ferroptosis via the Upregulation of HMOX-1 Expression

To further investigate the effects of 95% F1 on the expression of ferroptosis-related genes, we first conducted gene expression analysis and subsequently assessed HMOX-1 protein levels using cellular immunofluorescence experiments. The immunofluorescence results showed that, compared to the control group, HMOX-1 expression (green fluorescence) in NCI-H1975 cells was significantly upregulated in a dose-dependent manner with increasing concentrations of 95% F1 (Figure 4C,D). Concurrently, DAPI nuclear staining (blue fluorescence) revealed that although the intensity and distribution of the fluorescence signal remained relatively uniform across groups, the number of fluorescently labeled cells significantly decreased with higher drug concentrations, suggesting that 95% F1 may induce cell death.

Together with previous gene expression results, these findings further support the hypothesis that 95% F1 induces ferroptosis in lung cancer cells by activating HMOX-1-mediated pathways, enhancing ROS accumulation, and disrupting intracellular redox and iron homeostasis. This provides mechanistic evidence for the potential of 95% F1 as a ferroptosis-targeting anti-cancer agent.

These observations suggest that 95% F1 promotes ferroptosis in NCI-H1975 cells through the upregulation of HMOX-1, a critical oxidative stress-responsive enzyme. While HMOX-1 is known to protect cells against ROS-induced damage, in the context of ferroptosis, its upregulation can paradoxically facilitate cell death by contributing to iron release and oxidative imbalance.

### 2.4. Qualitative Analysis of the 95% F1 Fraction via UPLC-Q Exactive-MS

**Figure 5:**
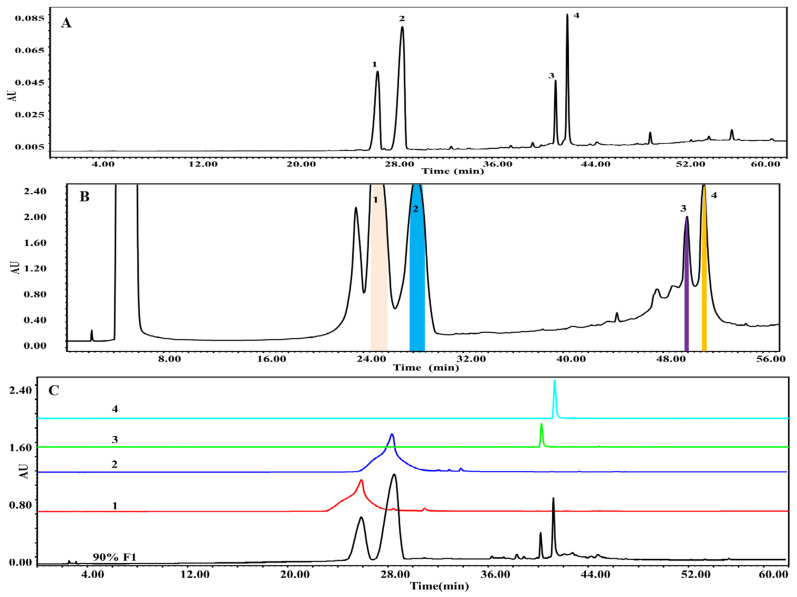
Semi-preparative HPLC separation of the 95% F1 fraction of *C. nitidissima* and chromatographic analysis of isolated compounds. (**A**) Analytical HPLC chromatogram of the 95% F1. (**B**) Semi-preparative HPLC chromatogram for the isolation of major components. (**C**) Purity assessment of isolated monomeric compounds via analytical HPLC. Mobile phase: methanol (solvent A) and water (solvent C); gradient elution: 0–5 min, 15% A; 5–50 min, linear gradient to 100% A; and 50–60 min, held at 100% A. Detection wavelength: 254 nm. Identified compounds: (1) isochlorogenic acid A, (2) isochlorogenic acid C, (3) apigenin, and (4) chrysin.

To further identify the bioactive compounds responsible for the observed pharmacological effects of the 95% F1 fraction and elucidate its material basis, a comprehensive component analysis was performed using UPLC-Q Exactive-MS. Based on database matching and literature comparison, four major compounds were identified: isochlorogenic acid A, isochlorogenic acid C, apigenin, and chrysin (Figure 5A). The structural information of these compounds is shown in Table 2.

Among them, apigenin and chrysin are flavonoids, while isochlorogenic acids A and C are derivatives of polyphenolic acids. These compounds are well known for their antioxidant properties and regulatory effects on cellular processes, consistent with the structural characteristics of ferroptosis inducers.

### 2.5. Molecular Docking and Molecular Dynamics Simulations to Screen Potential Bioactive Compounds

**Figure 6:**
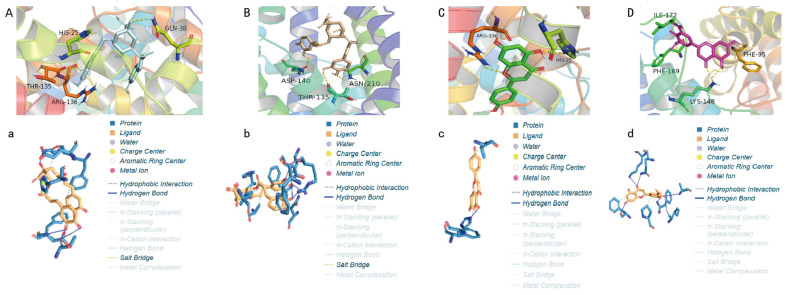
Two-dimensional and three-dimensional molecular docking models of active compounds from *C. nitidissima* with HMOX-1: (**A**,**a**) isochlorogenic acid A, (**B**,**b**) isochlorogenic acid C, (**C**,**c**) apigenin, and (**D**,**d**) chrysin.

Based on the UPLC-Q Exactive-MS analysis, the major identified compounds from the 95% F1 fraction were further evaluated for their potential bioactivity via molecular docking (Figure 6). HMOX-1, a key regulator of ferroptosis, was selected as the target protein. Docking simulations were performed to explore the binding interactions between HMOX-1 and isochlorogenic acid A, isochlorogenic acid C, apigenin, and chrysin. When binding energy values are negative, it suggests spontaneous binding between the ligand and the receptor, with more negative values indicating stronger binding affinity.

The results revealed that all four compounds exhibited strong interactions with critical residues of HMOX-1: apigenin with ARG136 and HIS25; chrysin with ILE172, PHE169, PHE95, and LYS148; isochlorogenic acid A with GLN38, HIS25, THR135, and ARG136; and isochlorogenic acid C with THR135, ASP140, and ASN210. Binding energy values were −7.8, −7.3, −8.4, and −8.4 kcal/mol, respectively, for these compounds in complex with the HMOX-1 structure (PDB ID: 3czy; Table 3). These results suggest good structural compatibility between ligands and the protein.

Among them, isochlorogenic acids A and C demonstrated the most favorable binding energies (−8.4 kcal/mol), forming multiple hydrogen bonds and polar interactions with the catalytic core of HMOX-1, indicating that they are the most promising lead candidates. In contrast, apigenin and chrysin, although with slightly lower affinities, may disrupt protein function through interactions with hydrophobic pockets. Differences in binding may be attributed to variations in hydroxyl group content, aromatic ring structures, and molecular flexibility, indicating that functional group conformation directly influences binding strength and stability. HMOX-1 contributes to ferroptosis by degrading heme and releasing Fe^2+^, which promote hydroxyl radical (•OH) formation via Fenton chemistry under oxidative stress, leading to lipid peroxidation. The binding sites of isochlorogenic acids overlapped with the catalytic region of HMOX-1, suggesting that they may modulate enzymatic activity or block substrate access. Apigenin and chrysin, which bind to allosteric sites, may induce conformational shifts that interfere with protein function. These compounds are likely responsible for the ferroptosis-inducing activity of the 95% F1 fraction in NCI-H1975 cells and may act as natural HMOX-1 regulators.

Molecular docking offers a visual and mechanistic explanation for ligand–target interactions and is advantageous in elucidating multi-component, multi-target mechanisms of traditional herbal medicine. However, docking alone is limited by its static, stoichiometric nature, which may lead to false-positive results. Therefore, we complemented this approach with MD simulations for dynamic validation.

**Figure 7:**
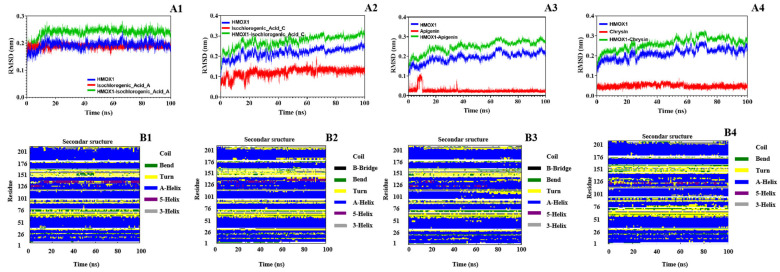
RMSD and secondary structure analysis of HMOX-1 in complex with active compounds from the 95% F1 fraction of *C. nitidissima* based on MD simulations. (**A1**–**A4**) RMSD plots of HMOX-1 complexes with (1) isochlorogenic acid A, (2) isochlorogenic acid C, (3) apigenin, and (4) chrysin, respectively. (**B1**–**B4**) Secondary structure evolution of HMOX-1 during the simulation in the presence of the same compounds, respectively.

Root mean square deviation (RMSD) values for the protein, ligands, and their complexes were calculated to assess conformational changes and binding stability throughout the simulation (Figure 7). RMSD is a key indicator of structural fluctuation; lower and more stable values reflect stable binding. During the early simulation phase (0–100 ns), fluctuations were observed as the systems adjusted, followed by stabilization. All complex RMSD values remained within a reasonable range (<0.3 nm), suggesting that ligand binding did not induce significant structural perturbation and reflected good compatibility.

**Figure 8:**
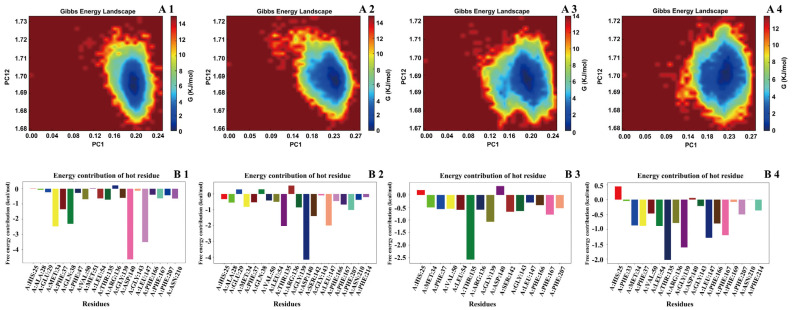
Gibbs free energy landscape and energy contribution of key residues in HMOX-1 binding with active compounds from the 95% F1 fraction of *C. nitidissima* based on MD simulations. (**A1**–**A4**) Gibbs free energy landscapes of HMOX-1 complexes with (1) isochlorogenic acid A, (2) isochlorogenic acid C, (3) apigenin, and (4) chrysin, respectively. (**B1**–**B4**) Per-residue energy contribution analysis identifying key “hot spot” residues involved in the binding of the same four compounds to HMOX-1.

Among the ligands, isochlorogenic acid A/C and chrysin exhibited minimal RMSD fluctuations, indicating stable binding conformations. Apigenin, due to its multiple hydroxyl groups and structural flexibility, showed more fluctuations before reaching equilibrium, suggesting potential reorientation during binding. Conformational analysis (Figure 8) using RMSD and the radius of gyration revealed distinct energy minima. Chrysin and isochlorogenic acids A and C maintained a single dominant low-energy state, reflecting stable protein conformations upon binding. In contrast, apigenin induced two distinct low-energy conformations, indicating multiple binding modes, possibly due to its smaller size and rigid structure.

Hydrogen bond analysis and the radial distribution function further clarified the interactions. Hydroxyl groups on the phenolic rings of all compounds formed stable hydrogen bonds with key residues such as Arg, His, and Tyr, which persisted throughout the simulation and contributed significantly to binding stability. Binding free energies (ΔG_total) between HMOX-1 and the four ligands were calculated using the molecular mechanics/generalized Born surface area (MM/GBSA) method (Table 4). The compounds exhibited the following total binding energies: isochlorogenic acid A: −42.38 kcal/mol, isochlorogenic acid C: −34.73 kcal/mol, chrysin: −27.56 kcal/mol, and apigenin: −20.71 kcal/mol. All ligands demonstrated strong binding affinity, with isochlorogenic acid A showing the most favorable total energy, indicating the highest binding stability. Van der Waals and electrostatic interactions were the major contributors, especially for isochlorogenic acid C, whose electrostatic interactions were enhanced, although partially offset by its high polar solvation energy. These findings suggest that the compounds bind reversibly and non-covalently to HMOX-1, leading to changes in local amino acid microenvironments and secondary structure via van der Waals forces, hydrogen bonds, and electrostatic interactions. Notably, upon ligand binding, the protein’s solvent-accessible surface area (SASA) and radius of gyration decreased, suggesting a more compact and stable conformation, rather than destabilization or unfolding.

Together, the MD simulation, hydrogen bonding network, conformational analyses, and binding energy calculations consistently demonstrated that the selected natural small molecules form energetically favorable complexes with HMOX-1. These results provide multi-level support for its role as a ferroptosis-inducing active constituent and a promising lead compound for developing HMOX-1-targeted natural therapeutic agents.

### 2.6. Isolation of Active Compounds Using Semi-Preparative HPLC

Based on the preliminary bioactivity screening results, the enriched active fractions were subjected to targeted isolation and purification using semi-preparative high-performance liquid chromatography (semi-preparative HPLC). The chromatographic conditions were systematically optimized, including the mobile phase composition, flow rate, gradient elution program, and detection wavelength. Ultimately, a methanol–water system was selected as the eluent, with the detection wavelength set at 254 nm, thereby allowing efficient gradient elution and the separation of the target compounds.

Through multiple rounds of preparative purification, four potential active monomeric compounds were successfully obtained (Figure 5B). HPLC analysis confirmed that each compound exhibited a purity greater than 90%. The chemical structures of the isolated compounds were further identified and validated using UPLC-Q Exactive-MS. The four purified compounds were identified as apigenin (90.03%), chrysin (90.16%), isochlorogenic acid A (95.83%), and isochlorogenic acid C (96.04%) (Figure 5C).

### 2.7. Pharmacological Validation of Monomeric Active Compounds

#### 2.7.1. Evaluation of Cytotoxic Activity

**Figure 9:**
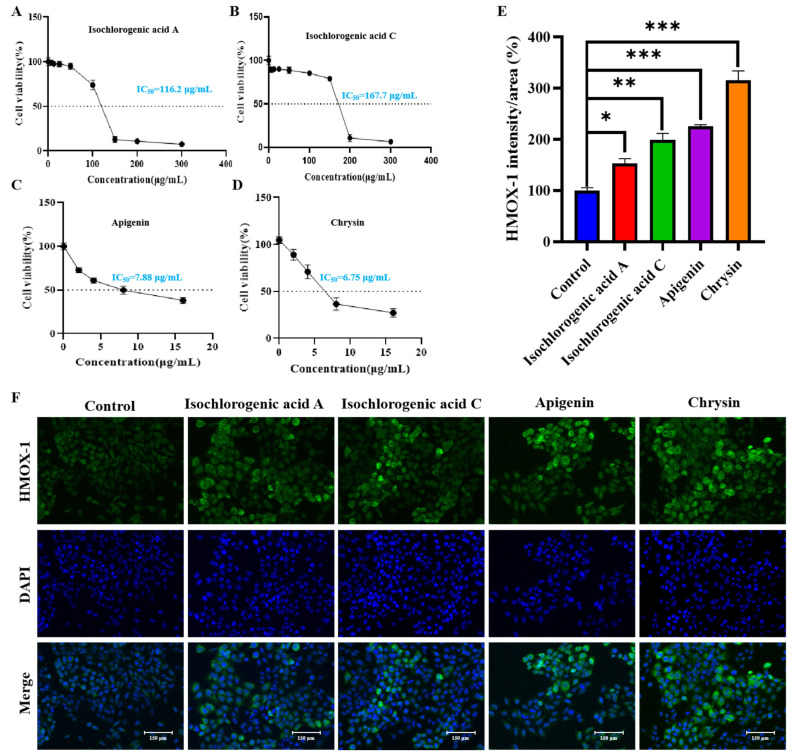
Evaluation of the cytotoxic effects and HMOX-1 expression in NCI-H1975 lung cancer cells treated with major active compounds from *C. nitidissima*. (**A**–**D**) Effect of isochlorogenic acid A, isochlorogenic acid C, apigenin, and chrysin on the viability of NCI-H1975 Cells. (**E**) Effects of different treatments on HMOX-1 expression levels. The bar graph shows the HMOX-1 intensity/area (%) in different treatment groups compared to the control group. (**F**) Immunofluorescence images showing HMOX-1 expression in NCI-H1975 cells following treatment with isochlorogenic acid A, isochlorogenic acid C, apigenin, and chrysin. Results are expressed as the mean ± SEM (*n* = 3). *: *p* < 0.05, **: *p* < 0.01, and ***: *p* < 0.0001 compared with the control group.

The cytotoxic effects of the four isolated monomeric compounds were assessed using a cell viability assay in NCI-H1975 lung cancer cells. The results revealed that chrysin and apigenin exhibited potent inhibitory effects, with IC_50_ values of 6.75 μg/mL and 7.88 μg/mL, respectively, indicating strong cytotoxicity. In contrast, isochlorogenic acid A and isochlorogenic acid C showed relatively weaker activity, with IC_50_ values of 116.2 μg/mL and 167.7 μg/mL, respectively (Figure 9A–D).

These findings suggest that chrysin and apigenin are likely the key bioactive compounds responsible for the anti-lung cancer effects observed in the 95% F1 fraction of *C. nitidissima*.

#### 2.7.2. Immunofluorescence-Based Evaluation of the Pharmacological Effects of Monomeric Compounds

Immunofluorescence analysis demonstrated that all four active compounds isolated from *C. nitidissima* leaves were capable of upregulating HMOX-1 expression in NCI-H1975 lung cancer cells to varying degrees (Figure 9E,F). Compared with the control group, cells treated with isochlorogenic acid A and isochlorogenic acid C showed a moderate increase in green fluorescence intensity corresponding to HMOX-1 expression, indicating a potential for inducing HMOX-1 at the protein level.

Notably, apigenin and chrysin induced markedly stronger HMOX-1 expression, with chrysin exhibiting the most intense fluorescence signal. These results are consistent with their previously observed high cytotoxic activity.

The immunofluorescence images revealed that HMOX-1 fluorescence was predominantly localized in the cytoplasmic region, with some redistribution toward the perinuclear area in certain treatment groups. This pattern may reflect the involvement of HMOX-1 in cellular oxidative stress responses. However, further investigation is required to elucidate the precise molecular mechanisms underlying this subcellular localization.

Importantly, these findings are in strong agreement with the PCR Array data showing significant upregulation of HMOX-1 mRNA expression. Together, they indicate that the active components of *C. nitidissima* can promote HMOX-1 expression at both the transcriptional and translational levels, thereby activating ferroptosis pathways. This provides compelling molecular evidence supporting the therapeutic potential of these natural compounds as anti-tumor agents.

## 3. Discussion

In the research and development of natural products and new drugs, the effective extraction, separation, and purification of bioactive compounds are crucial steps for elucidating pharmacological foundations [22,23,24]. Ethanol, as an extraction solvent, efficiently dissolves a wide range of polar and semi-polar compounds while maintaining a low toxicity profile. This makes it essential for the comprehensive isolation of potential anti-cancer components. Therefore, in this study, 75% ethanol was selected for extracting bioactive compounds from *C. nitidissima* leaves. In this study, polyamide column chromatography was combined with the tracking of active compounds and semi-preparative HPLC. By leveraging the pore structure and molecular sieving effect of polyamide resin [25,26,27], multiple active fractions were successfully isolated from the extract of *C. nitidissima* leaves (10% F2, 30% F1, 30% F2, 70% F1, 70% F2, 95% F1, and 95% F2). Among these, the 95% F1 fraction exhibited significant inhibitory effects on the NSCLC cell line NCI-H1975.

Certain traditional Chinese medicinal plants, such as the ethanol extract of *Ruta graveolens*, have been reported to inhibit the proliferation of NCI-H1975 cells by approximately 50% at a concentration of 53.3 μg/mL [28]. In contrast, the IC_50_ value of the *C. nitidissima* leaf extract in our study was 27.97 μg/mL, and that of the 95% F1 was as low as 10.95 μg/mL, indicating significantly stronger cytotoxic activity against the same NSCLC model. It is worth noting that *C. nitidissima*, as a dual-purpose plant used in both food and medicine, has been widely consumed as tea and applied in traditional Chinese medicine for heat-clearing, detoxification, and anti-inflammatory purposes. Its extract has generally exhibited low toxicity in both animal and cellular studies [29]. This study primarily focused on evaluating the anti-cancer effects of the *C. nitidissima* leaf extract through ferroptosis induction in NSCLC cells. Based on its long history of traditional use, the extract is presumed to have low toxicity toward normal cells. However, given its potential for therapeutic application, it is crucial to further assess its selectivity and safety. Future studies will include toxicity evaluations in normal lung cells to determine the therapeutic window and potential side effects of the *C. nitidissima* leaf extract and its active constituents. These findings will provide essential scientific evidence for its development as a clinically relevant anti-cancer agent.

Compared with traditional anti-cancer drugs, compounds that induce ferroptosis possess unique advantages, such as efficacy against drug-resistant cancer cells and lower systemic toxicity. In this study, treatment with the 95% F1 fraction significantly modulated the typical molecular markers of ferroptosis: increased levels of ROS and MDA, along with decreased levels of GSH. These changes are hallmark features of ferroptosis, indicating the occurrence of oxidative stress and lipid peroxidation within cells. ROS are by-products of cellular metabolism, and when present in excess, they can cause oxidative damage to cellular components. MDA, a product of lipid peroxidation, serves as a biomarker of oxidative stress and cellular damage. The reduction in GSH levels indicates the impairment of the cellular antioxidant defense system, rendering cells more susceptible to oxidative damage and subsequently leading to ferroptosis. TEM observations of the mitochondrial ultrastructure revealed that treatment with the 95% F1 fraction caused mitochondrial shrinkage, increased membrane density, and a loss of or reduction in cristae. The disruption of mitochondrial integrity and function may be caused by oxidative stress-induced lipid peroxidation of mitochondrial membrane lipids, resulting in the loss of mitochondrial membrane potential, impaired energy metabolism, and ultimately cell death, consistent with the morphological features of ferroptosis. Collectively, these findings demonstrate the cellular basis of ferroptosis induction by the 95% F1 fraction.

Moreover, the upregulation of ROS and MDA levels, along with the downregulation of GSH levels, indicates a positive correlation between ROS accumulation and lipid peroxidation and a negative correlation between GSH levels and ROS generation. This interplay highlights the central role of oxidative stress in driving ferroptosis and underscores the importance of maintaining cellular redox balance. The observed mitochondrial structural changes further support the notion that oxidative stress-induced mitochondrial membrane damage is a key event in the ferroptosis process. Furthermore, other botanicals like Curcuma longa and Ginkgo biloba exert anti-cancer effects primarily through apoptosis or autophagy pathways [30,31], and the *C. nitidissima* leaf extract induces ferroptosis in NSCLC cells by modulating intracellular ROS, MDA, and GSH levels and upregulating HMOX-1. This highlights a mechanistic advantage and underscores the potential of *C. nitidissima* as a ferroptosis-targeted natural anti-cancer agent.

In summary, the 95% F1 fraction induces ferroptosis in NCI-H1975 cells by upregulating ROS and MDA levels and downregulating GSH levels, resulting in mitochondrial structural damage and cell death. These findings not only elucidate the molecular and cellular mechanisms underlying ferroptosis induction by the 95% F1 fraction but also highlight the significance of each measured parameter and its interrelatedness in the context of ferroptosis. Compared to other plant extracts, the distinctive ability of the *C. nitidissima* extract to induce ferroptosis further underscores its potential as a promising and novel anti-cancer therapeutic agent.

At the molecular mechanism level, we explored the molecular mechanisms underlying ferroptosis induction by the 95% F1 fraction from the *C. nitidissima* leaf extract by analyzing the expression profile of ferroptosis-related genes in NCI-H1975 cells. The results indicated that the 95% F1 fraction significantly affected the expression of multiple ferroptosis-related genes, with the remarkable upregulation of HMOX-1 being particularly noteworthy. HMOX-1 is a key enzyme involved in the rate-limiting step of heme degradation, generating carbon monoxide, ferrous iron, and biliverdin. These products not only possess antioxidant and anti-inflammatory properties that protect cells from oxidative stress damage but may also promote ferroptosis by accelerating the release of Fe^2^⁺ and the lipid peroxidation chain reaction. Moreover, the upregulation of other ferroptosis-related genes, such as CHAC1, NOX1, NOX3, FTMT, CDO1, and MAP1LC3B, further enhanced cellular sensitivity to oxidative stress and facilitated the occurrence of ferroptosis. The synergistic action of these genes suggests that the 95% F1 fraction regulates iron metabolism, lipid oxidation, and redox homeostasis through multiple pathways, thereby effectively inducing ferroptosis. Immunofluorescence confirmed the pivotal role of HMOX-1 in this study, which is highly consistent with the latest research hotspots in ferroptosis. HMOX-1 not only plays a central role in the process of ferroptosis, but its upregulation has also been demonstrated to confer significant protective effects in various diseases, including cardiovascular diseases, inflammatory conditions, and viral infections [32,33,34]. These findings highlight its potential as a highly promising therapeutic target. Therefore, pharmacological interventions to upregulate HMOX-1 expression could not only effectively induce ferroptosis but also provide new therapeutic strategies for multiple diseases. In summary, the key role of HMOX-1 in ferroptosis and its pleiotropic effects make it a highly promising research target. In-depth investigation of its regulatory mechanisms and functions may offer new strategies and therapeutic targets for the treatment of diseases such as lung cancer.

Computer-aided drug design, particularly molecular docking and MD simulations, has emerged as a powerful tool for identifying and optimizing potential bioactive compounds [35,36,37]. Molecular docking can effectively predict the binding modes of small molecules with target proteins and evaluate key interactions such as hydrogen bonds and hydrophobic interactions, thereby efficiently screening candidate molecules with the optimal binding conformations [38,39]. In addition, MD simulations can dynamically monitor the stability of ligand–receptor complexes in simulated solvent environments and calculate binding free energies using methods such as MM/GBSA, thereby improving the accuracy and reliability of screening methods [40]. Compared with traditional experimental screening methods, computational simulations significantly reduce the time and cost while allowing for structural modifications to optimize the stability and specificity of molecular binding [41].

By leveraging UPLC-Q Exactive MS in combination with molecular docking and MD simulations, we identified isochlorogenic acid A, isochlorogenic acid C, apigenin, and chrysin as core bioactive compounds. Although these compounds are generally known for their antioxidant properties, studies have shown that polyphenols, particularly multi-hydroxylated flavonoids, can disrupt the mitochondrial electron transport chain, leading to electron leakage and excess ROS production. They may also react with GSH or inhibit its regeneration, thereby depleting cellular antioxidant reserves. These effects may cause polyphenols to shift from antioxidants to pro-oxidants under specific conditions, promoting intracellular ROS accumulation [42,43]. In our study, within the oxidative stress-enriched tumor cell environment, the phenolic constituents in the 95% F1 fraction likely underwent this role transition, contributing to further ROS elevation and reaching a cytotoxic threshold that ultimately triggered ferroptosis and other oxidative stress-related cell death pathways.

HMOX-1 is a key enzyme responsible for heme degradation and can promote ferroptosis by releasing Fe^2^⁺. Apigenin and chrysin formed stable interactions with HMOX-1, with binding energies of −7.8 and −7.3 kcal/mol, respectively, and MD simulations revealed stable binding site conformations. These interactions are not only stable but also facilitate the release of Fe^2^⁺, thereby accelerating the lipid peroxidation process—a core mechanism of ferroptosis—ultimately leading to a significant increase in ROS. As such, these compounds play a pivotal role in the induction of ferroptosis and further emphasize the critical role of HMOX-1 in this process, providing an important molecular target for the development of natural ferroptosis modulators targeting HMOX-1.

Subsequently, high-purity separation of the four core bioactive components was achieved using semi-preparative HPLC based on reversed-phase HPLC [37,38,39], and their effects were confirmed in vitro. Apigenin and chrysin were found to effectively induce ferroptosis in NCI-H1975 cells, with their cytotoxic activities being closely related to the upregulation of HMOX-1. Unlike previous studies on traditional Chinese medicine that primarily focused on the inhibition of GPX4/SLC7A11 in ferroptosis pathways, this study highlights the key role of HMOX-1 and emphasizes the complete chain mechanism of “promoting iron ion release—disrupting iron homeostasis—uncontrolled oxidative stress response—cell death”. This approach lays the groundwork for the development of natural ferroptosis modulators targeting HMOX-1 by providing lead compounds and a molecular basis.

Although this study systematically established the molecular mechanisms and material basis for ferroptosis induction by the *C. nitidissima* leaf extract, the current findings are primarily based on in vitro cell models and molecular simulations. Future work will consider validating the in vivo ferroptosis-inducing potential and safety of the *C. nitidissima* leaf extract and its monomer components in mouse xenograft models, gene-edited cells, and patient-derived samples. Additionally, further preclinical studies will help assess the pharmacokinetic properties and potential toxicity of these compounds, providing a scientific basis for future clinical applications.

In summary, *C. nitidissima*, a plant with multiple natural bioactive components and controllable costs, has shown great potential in cancer prevention and treatment. This study not only provides theoretical support and experimental evidence for the innovative transformation of local traditional Chinese medicine and edible resources but also offers strong support for the development of natural drugs targeting HMOX-1, potentially opening up new avenues for the treatment of NSCLC and other diseases.

## 4. Materials and Methods

### 4.1. Extraction and Preparation of Bioactive Fractions from C. nitidissima Leaves

The dried leaves of *C. nitidissima* (5.0 kg) were cut into small pieces and soaked in 75% ethanol at a solid–liquid ratio of 1:25 (*w*/*v*) for 30 min at room temperature. The mixture was then reflux-extracted for 2.0 h, and this procedure was repeated twice. The combined extracts were filtered, concentrated under reduced pressure using a rotary evaporator until a thick syrup was obtained, pre-frozen at −80 °C, and subsequently freeze-dried to yield a lyophilized ethanolic extract powder.

An aliquot (480 g) of the ethanolic extract powder was dissolved in methanol, mixed thoroughly with 650 g of polyamide resin, and allowed to stand until complete evaporation of the organic solvent. The dried mixture was evenly loaded onto a pretreated polyamide chromatographic column, ensuring the resin bed remained submerged below the solvent surface to prevent cracking. Gradient elution was performed sequentially with distilled water, followed by 10%, 30%, 70%, and 95% ethanol, using 3.0 column volumes (3.0 BV) for each gradient at a flow rate of 1.0 BV/h. Each fraction obtained was collected, freeze-dried, weighed, and stored for subsequent bioactivity screening and chemical composition analyses.

### 4.2. Cell Lines and Culture

The human NSCLC cell lines A549, NCI-H661, and NCI-H1975 were provided by the Guangxi Key Laboratory of Efficacy Study on Chinese Materia Medica. Cells were cultured in a medium supplemented with 10% fetal bovine serum (FBS, 1939723, Gibco, Waltham, MA, USA) and maintained at 37 °C in a humidified incubator with 5% CO_2_. The culture medium was regularly refreshed, and cells were passaged during the logarithmic growth phase to ensure optimal cell viability and experimental consistency.

### 4.3. Evaluation of Ferroptosis Induction and Anti-NSCLC Activity

#### 4.3.1. Cell Culture and Viability Assessment

The sensitivity of three NSCLC cell lines (A549, NCI-H661, and NCI-H1975) to *C. nitidissima* ethanolic extracts was evaluated using the Cell Counting Kit-8 assay (CCK-8; BCCK0100, Bioswamp, Osaka, Japan). Cells in the logarithmic growth phase were seeded into 96-well plates at a density of 3 × 10^4^ cells/mL. When cells reached approximately 30–40% confluence, they were treated with varying concentrations (25–150 μg/mL) of the ethanolic extracts for 48 h. After treatment, 10 μL of the CCK-8 reagent was added to each well, and the cells were incubated for an additional 2.0 h. Absorbance was measured at 450 nm using a microplate reader (Multiskan, Thermo Fisher Scientific, Waltham, MA, USA). The half-maximal inhibitory concentration (IC_50_) values were calculated using GraphPad Prism 8.0.2. The most sensitive cell line (NCI-H1975) was selected for subsequent experiments.

Similarly, fractions obtained from polyamide column chromatography were screened against NCI-H1975 cells using the same protocol, and the most biologically active fraction (95% F1) was identified. To further investigate the involvement of ferroptosis in 95% F1-induced cytotoxicity, NCI-H1975 cells were divided into three groups: a control group, 95% F1-treated groups (2.0, 4.0, 8.0, and 16 μg/mL), and a co-treatment group receiving both 95% F1 and the ferroptosis inhibitor Ferrostatin-1 (Fer-1, 5.0 μmol, HY-100759, MedChemExpress, Monmouth Junction, NJ, USA). After 48 h of incubation, cell viability was assessed using the CCK-8 assay.

#### 4.3.2. Assessment of Oxidative Stress and Iron Metabolism Markers

This study aimed to evaluate the impact of fraction 95% F1 on oxidative stress and ferroptosis-related markers in NCI-H1975 cells. Intracellular levels of reactive oxygen species (ROS, S0033S, Beyotime, Haimen, China), glutathione (GSH, BC1175, Solarbio, Beijing, China), malondialdehyde (MDA, BC0025, Solarbio, Beijing, China), and ferrousion (Fe^2+^, BC5315, Solarbio, Beijing, China) were measured. Logarithmic-phase NCI-H1975 cells were digested with trypsin, adjusted to a density of 5.0 × 10^4^ cells/mL, and seeded in 6-well plates with 2.0 mL per well. Cells were cultured overnight at 37 °C in a humidified incubator with 5% CO_2_. Subsequently, cells were treated with 95% F1 at concentrations of 4.0, 8.0, and 16 μg/mL for 48 h. For ROS detection, after removing the culture medium, cells were incubated with 1.0 mL of the serum-free medium containing 10 μmol/L DCFH-DA in the dark at 37 °C for 20 min, washed three times with PBS, and analyzed using flow cytometry. GSH, MDA, and Fe^2+^ were measured after lysing cells with the appropriate extraction buffers, collecting supernatants post-centrifugation, and assessing absorbance at 410 nm (GSH), 523 nm and 600 nm (MDA), and 510 nm (Fe^2+^), respectively, using a microplate reader. The results comprehensively evaluated the effect of 95% F1 on oxidative stress and iron metabolism.

#### 4.3.3. Transmission Electron Microscopy for Mitochondrial Morphology

Logarithmic-phase NCI-H1975 cells were routinely digested and seeded in 25 cm^2^ culture flasks at a density of 2.0 × 10^6^ cells/mL and incubated at 37 °C with 5% CO_2_ overnight. The next day, cells were treated with 8.0 μg/mL of fraction 95% F1 or an equal volume of the solvent as the control for 48 h. The supernatant was discarded, and cells were harvested via trypsin digestion, and pellets were fixed in an electron microscopy fixative solution (P1126, Solarbio) at room temperature for 2.0 h in the dark. Subsequent steps included dehydration, infiltration, embedding, sectioning, and staining. Mitochondrial ultrastructural changes were examined, and images were captured using transmission electron microscopy (JEM-1400FLASH, JEOL, Tokyo, Japan).

#### 4.3.4. PCR Array Analysis of Ferroptosis-Related Genes

NCI-H1975 cells in logarithmic growth were seeded into 25 cm^2^ culture flasks at 1.0 × 10^5^ cells/mL and treated with fraction 95% F1 for 48 h. Post-treatment, cell pellets were collected. Total RNA was extracted using the Eastep Super reagent (LS1040, Promega, Madison, WI, USA), reverse transcribed to cDNA with the GoScript Reverse Transcription System (A5001, Promega), and amplified using the GoTaq^®^ qPCR Master Mix (A6001, Promega). Real-time PCR was performed on a Roche LightCycler^®^ 96 (Tgradient, Biometra, Göttingen, Germany), and relative gene expression changes were calculated using the 2^−ΔΔCt^ method. Primers for ferroptosis-related genes were obtained from a ferroptosis PCR array kit (WC-mRNA0271-H, Wcgene Biotechnology, Shanghai, China). The list of target genes is shown in Table S1.

#### 4.3.5. Immunofluorescence Assay for the HMOX-1 Protein

Based on the PCR Array results, the key regulatory protein HMOX-1 was selected for evaluation via immunofluorescence. After treatment with fraction 95% F1 for 48 h, NCI-H1975 cells were fixed with 4% paraformaldehyde (70120900, Biosharp, Tallinn, Estonia) for 15 min at room temperature, permeabilized with 0.1% Triton X-100 (A110694-0100, Sigma Sangon Biotech, Shanghai, China) for 10 min, and blocked for 30 min. Cells were incubated overnight at 4.0 °C with an anti-HMOX-1 primary antibody (10701-1-AP, Proteintech, Singapore, 1:500), followed by incubation with the Multi-rAb CoraLite^®^ Plus 488 Goat Anti-Rabbit Recombinant Secondary Antibody (H+L) (RGAR002, Proteintech, 1:500) at room temperature for 1.0 h. Finally, cells were counterstained with the DAPI antifade mounting medium (S2100, Solarbio), and images were captured using a fluorescence microscope (EVOS^™^ M5000 Imaging System, Thermo Fisher Scientific, Waltham, MA, USA).

### 4.4. UPLC-Q Exactive MS Analysis of Active Fractions

The potential bioactive components in fraction 95% F1 from *C. nitidissima* leaves were analyzed using an ACQUITY UPLC I-Class HF system coupled with UPLC-Q Exactive MS (Thermo Fisher Scientific). The analysis was performed using an ACQUITY UPLC^®^ BEH C_18_ column (1.7 µm, 2.1 × 50 mm). The mobile phase consisted of methanol (B) and either water or a 0.1% formic acid aqueous solution (A), with a flow rate of 0.3 mL/min. Gradient elution was programmed as follows: 0–2 min, 2% B; 2–10 min, 2–98% B; and 10–13 min, held at 98% B.

Mass spectrometry was carried out using an electrospray ionization (ESI) source operated in both positive and negative ionization modes. In the positive ion mode, the source voltage was set to 3.2 or 3.5 kV, while in the negative ion mode, it was set to 2.8 or 3.0 kV. The capillary temperature was maintained at 320 °C, and the source heater temperature was maintained at at 350 °C. The sheath gas flow rate was 40 arbitrary units; the auxiliary gas flow rate was 10 arbitrary units, and the sweep gas flow rate was 0.

Data were acquired in Full MS/dd-MS^2^ mode, with a resolution of 70,000 for MS and 17,500 for MS/MS. The normalized collision energy (NCE) was set at 29. Depending on the ionization mode, a 0.1% formic acid aqueous solution was used as mobile phase A for the positive ion mode, while pure water was used as mobile phase A for the negative ion mode. Methanol was used as mobile phase B in both cases. Data were processed using the Thermo Xcalibur software and identified through comparative analysis using the LutMet-CM 1.0, Herb, and LuMet-animal databases.

### 4.5. Molecular Docking and Molecular Dynamics Simulation

Molecular docking was performed using Autodock Tools 1.5.6 to screen the identified active compounds against the core target protein, HMOX-1 (PDB ID: 3czy). The 3D structures of four active constituents were prepared using ChemDraw3D 2.0, optimized with Autodock 4.2 Ligand tools, and docked to the prepared protein structure using a modified genetic algorithm. Docking parameters included hydrogen bonding, ionic interactions, and hydrophobic and hydrophilic interactions. Binding energies were calculated, with lower energies indicating more stable binding. Upon completion of docking, visual analysis was conducted using Pymol and Discovery Studio Visualizer 2022.

Center_x = 11.789; center_y = −10.496; center_z = 18.63.

Size_x = 54; size_y = 70; size_z = 76.

MD simulations were performed using Gromacs 2022.3 with the Amber99sb-ildn force field and the Tip3p water model. Systems were neutralized with sodium ions, energy-minimized, equilibrated under NVT and NPT conditions, and subjected to a 100 ns MD simulation at 2.0 fs per step. RMSD, RMSF, the radius of gyration, and free energy profiles (MM/GBSA) were analyzed to assess the stability and interaction mechanisms of protein–ligand complexes. Upon completion of the simulations, trajectory analysis was carried out using the software’s built-in tools, and final data were calculated.

### 4.6. Semi-Preparative HPLC Isolation and Purity Assessment

Guided by computer-aided virtual screening results, the active chemical constituents with strong binding affinity toward the potential ferroptosis target HMOX-1 were selectively isolated and purified using semi-preparative HPLC. By optimizing the mobile phase composition and the gradient elution program, efficient separation of the target active compounds was achieved. Each chromatographic fraction was subsequently collected and analyzed via analytical HPLC to determine its purity, ensuring the reliability and reproducibility of subsequent pharmacological validation experiments.

Chromatographic separation was performed using gradient elution with methanol (A) and water (C) as the mobile phases. The gradient program was as follows: 0–5 min, 15% A; 5–50 min, a linear increase to 100% A; and 50–60 min, maintained at 100% A. Detection wavelengths were set at 254 nm.

Analytical HPLC was conducted using gradient elution with methanol (A) and water (C) as the mobile phases. The gradient conditions were as follows: 0–5 min, 5% A; 5–50 min, linearly increased to 100% A; and 50–60 min, held at 100% A. The detection wavelength was set at 254 nm.

### 4.7. Cytological Pharmacological Validation of Active Compounds

#### 4.7.1. Cell Viability Assay

The cell viability of the isolated compounds was assessed according to methods described in Section 4.3.1.

#### 4.7.2. Immunofluorescence Assay for Active Compounds

To further investigate the relationship between the screened monomeric active compounds and the regulatory mechanisms of ferroptosis, immunofluorescence analysis was performed to assess the expression of the HMOX-1 protein in NCI-H1975 cells following 48 h of treatment with the target monomeric compounds, as described in Section 4.3.5.

### 4.8. Statistical Analysis

Data are expressed as the mean ± standard deviation ($\bar{x}\pm s$). Statistical analysis and plotting were performed using GraphPad Prism 8.0.2. Differences among groups were analyzed using one-way ANOVA. Statistical significance was set at *p* < 0.05 (*), <0.01 (**), and <0.0001 (***). *p* > 0.05 indicated no significant difference.

## 5. Conclusions

This study elucidates the mechanism by which *C. nitidissima* exerts its anti-lung cancer effects through the induction of ferroptosis and identifies key active components—apigenin, chrysin, and isochlorogenic acids A and C—that target HMOX-1, a critical molecular player in ferroptosis. These findings provide a theoretical basis for the development of natural lead compounds targeting ferroptosis in the treatment of NSCLC. Our findings not only provide valuable lead compounds for the development of novel anti-cancer strategies based on ferroptosis but also offer theoretical support for the exploration of such approaches.

Moreover, our results align with the growing interest in harnessing natural products to modulate ferroptosis as a therapeutic strategy for cancer. They underscore the importance and utility of computer-aided screening in identifying interactions between natural compounds and their molecular targets, thereby enhancing the scientific rationale for the application of traditional Chinese medicinal plants, such as *C. nitidissima*, in oncology.

## Figures and Tables

**Table 1:** Weight, yield, and cytotoxic activity (IC_50_) of different polarity fractions from *C. nitidissima* extract against NCI-H1975 lung cancer cells. $\bar{x}\pm SEM$ (*n* = 3).

Sample	Weight (g)	Yield (%)	IC_50_ (μg/mL)
10% F2	16.02	3.34	——
30% F1	9.90	1.88	——
30% F2	23.11	4.81	130.825 ± 5.166
70% F1	75.96	15.83	47.6175 ± 5.459
70% F2	130.48	27.18	15.1625 ± 0.732
95% F1	10.21	2.13	10.95 ± 0.170
95% F2	64.78	13.49	13.565 ± 0.339

**Table 2:** Identification of major active compounds from the 95% F1 fraction of *C. nitidissima* using UPLC-Q-Exactive MS/MS in negative ion mode.

No.	Compound	Formula	Structure	*m*/*z * (Theoretical)	*m*/*z* (Observed)	Mass Error (ppm)	*m*/*z* (MS/MS) Fragments
1	Isochlorogenic acid A	C_25_H_24_O_12_	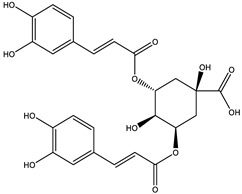	515.1184 [M-H]^−^	515.1159 [M-H]^−^	−4.85	353.1083, 335.0903, and 191.0564
2	Isochlorogenic acid C	C_25_H_24_O_12_	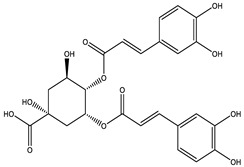	515.1184 [M-H]^−^	515.1169 [M-H]^−^	−2.91	353.0969, 191.0546, and 173.0445
3	Apigenin	C_15_H_10_O_5_	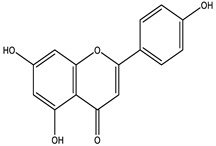	269.0450 [M-H]^−^	269.0449 [M-H]^−^	−0.37	151.0303, 117.0802, and 107.1213
4	Chrysin	C_15_H_10_O_4_	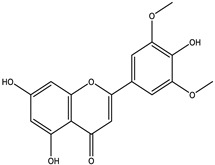	253.0493 [M-H]^−^	253.0502 [M-H]^−^	+3.56	209.0703, 143.0504, and 107.0153

**Table 3:** Molecular docking analysis of potential HMOX-1 inhibitors and the 3czy protein in *C. nitidissima*.

NO.	Ligand Name	Docking Energy (kcal/mol)	Interaction Force	Combination Method	Major Amino Acid Residues
1	Isochlorogenic acid A	−8.4	Hydrophobic interaction, salt bridge, and hydrogen bonding	Non-covalent binding	GLN38, HIS25, THR135, and ARG136
2	Isochlorogenic acid C	−8.4	Hydrophobic interaction, salt bridge, and hydrogen bonding	Non-covalent binding	THR135, ASP140, and ASN210
3	Apigenin	−7.8	Hydrophobic interaction and hydrogen bonding	Non-covalent binding	ARG136 and HIS25
4	Chrysin	−7.3	Hydrophobic interaction and hydrogen bonding	Non-covalent binding	ILE172, PHE169, PHE95, and LYS148

**Table 4:** MM/GBSA computational combination free.

NO.	Ligand Name	Formula	Binding Free Energy (kcal/mol)						
			ΔE_vdw_	ΔE_eel_	ΔEGB	ΔESURF	ΔG_gas_	ΔG_solv_	ΔTOTAL
1	Isochlorogenic acid A	C_25_H_24_O_12_	−49.46	−40.45	55.11	−7.57	−89.92	47.54	−42.38
2	Isochlorogenic acid C	C_25_H_24_O_12_	−39.00	−71.05	82.25	−6.93	−110.05	75.32	−34.73
3	Apigenin	C_15_H_10_O_5_	−28.27	−18.40	30.17	−4.22	−46.67	25.95	−20.71
4	Chrysin	C_15_H_10_O_4_	−30.08	−14.42	30.25	−5.31	−52.50	24.93	−27.56

## Data Availability

The original contributions presented in this study are included in the article/Supplementary Materials. Further inquiries can be directed to the corresponding author.

## Supplementary Materials

The following supporting information can be downloaded at: https://www.mdpi.com/article/10.3390/ph18060824/s1, Table S1: List of ferroptosis-related genes.
