# Supplementary material for: Identification of HMOX-1-Targeting Natural Compounds in Camellia nitidissima Chi for NSCLC Therapy: Integrating Bioassay and In Silico Screening Approaches
Source: Pharmaceuticals (Basel). 2025 May 30;18(6):824. doi: 10.3390/ph18060824 (PMC12196505; doi:10.3390/ph18060824)
Supplement: Supplementary file 1 [file pharmaceuticals-18-00824-s001.zip › pharmaceuticals-3645301-supplementary.pdf]

Table S1. List of ferroptosis-related genes.

NO.	A	B	C	D	E	F	G	H
1	ACO1	ACSL4	AKR1B1	AKR1B10	AKR1C1	ALDH1A1	ALOX15	ATF4
2	ATG5	ATP5G3	BBC3	BECN1	BRAF	BRD4	CA9	CARS1
3	CDO1	CHAC1	ClSD1	ClSD2	CP	CS	CYBB	DMT1
4	DPP4	ELAVL1	EMC2	EPRS	FTH1	FTL	FTMT	GCLC
5	GCLM	GLS2	GOT1	GPX4	GSS	GSTA1	GSTP1	HAMP
6	HARS	HEPH	HEF	HMOX1	HMOX2	HRAS	HSF1	HSPB1
7	IREB2	KEAP1	KRAS	LOX	LPCAT3	MAP1LC3A	MAP1LC3B	ACTB
8	NCOA4	NFE2L2	NOX1	NOX3	NOX4	NQO1	NRAS	GAPDH
9	PCBP1	PCBP2	PPARG	PRDX	PRNP	PTGES2	RPL8	HPRT1
10	SAT2	SLC1A5	SLC39A14	SLC39A8	SLC3A2	SLC40A1	SLC7A11	18S
11	STEAP3	STIM1	TF	TFR1	TFR2	TP53	TXNRD1	NTC
12	VDAC2	VDAC3	MAP1LC3C	PANX2	SAT1	SQSTM1	USP7	NTC
